# The Effect of Self-Rehabilitation on Physical Fitness in Adults with Hematological Malignancies (HMs) Undergoing Hematopoietic Stem Cell Transplantation (HSCT)

**DOI:** 10.3390/cancers18152421

**Published:** 2026-07-28

**Authors:** Michał Chmielewski, Agnieszka Szeremet, Paula Jabłonowska-Babij, Maciej Majcherek, Anna Czyż, Tomasz Wróbel, Iwona Malicka

**Affiliations:** 1 Active Recovery, Non-Profit Foundation, 53-019 Wroclaw, Poland; 2 Department and Clinic of Hematology, Cellular Therapies and Internal Medicine, Wroclaw Medical University, 50-367 Wroclaw, Poland; 3 Department of Nursing, Faculty of Nursing and Midwifery, Wroclaw Medical University, 51-618 Wroclaw, Poland; 4 Department of Physiotherapy, Wroclaw University of Health and Sport Sciences, 51-612 Wroclaw, Poland

**Keywords:** hematologic neoplasms, hematopoietic stem cell transplantation, exercise therapy, physical fitness

## Abstract

**Background**: Hematologic malignancies (HMs) represent a growing global health burden, and HSCT remains a cornerstone of treatment for many HMs. Despite improvements in survival, transplantation-related physical deconditioning is a persistent clinical challenge, and evidence supporting structured inpatient exercise interventions is scarce. **Objectives:** To evaluate the effects of a self-directed aerobic exercise program on a cycle ergometer in inpatients under isolation precautions related to HSCT. **Methods:** In this single-center prospective, non-randomized controlled interventional study, 73 patients were enrolled in an intervention group (*n* = 43) performing daily aerobic exercise on a cycle ergometer, or a control group (*n* = 30) receiving standard care. Physical performance was assessed at baseline and at discharge using the 6-min walk test (6 MWT), Timed Up and Go test (TUG), and 30-s chair stand test (30 s-CST). **Results**: Significant group-by-time interactions were observed for the TUG (F(1,71) = 10.35; *p* = 0.001; ηp^2^ = 0.13) and the 30 s-CST (F(1,71) = 21.61; *p* < 0.0001; ηp^2^ = 0.23). No significant interaction was detected for the 6 MWT (F(1,71) = 2.01; *p* = 0.16; ηp^2^ = 0.03), although the decline in walking distance was smaller in the intervention group than in the control group (−4.1% vs. −10.5%). Post hoc analyses demonstrated significant within-group deterioration only in the control group. **Conclusions:** Self-directed aerobic exercise during inpatient isolation may mitigate physical deconditioning in HSCT recipients, supporting its integration into routine care.

## Simple Summary

Patients undergoing hematopoietic stem cell transplantation (HSCT) often experience significant physical deconditioning during prolonged hospitalization in protective isolation. Although exercise may help reduce functional decline, supervised rehabilitation is not always feasible in transplant units. In this study, we evaluated a simple self-directed aerobic exercise program using a cycle ergometer in hospitalized HSCT recipients. Patients participating in the exercise program demonstrated better preservation of functional mobility and less deterioration in physical performance compared with patients receiving standard care. These findings suggest that simple, low-cost and feasible exercise interventions may be a useful component of supportive care during HSCT.

## 1. Introduction

Hematologic malignancies (HMs) represent a major and growing challenge in contemporary oncology and public health [1]. According to the most recent data from the Global Cancer Observatory, HMs accounted for 6.6% of total cancer cases and 7.2% of total cancer-related deaths worldwide in 2022 [2], and their global burden has risen steadily over the past three decades. The absolute number of incident cases has increased continuously since 1990, while age-standardized death rates for all major subtypes have declined [3]. This epidemiological paradox reflects both advances in risk stratification and disease monitoring and, more substantially, a therapeutic revolution driven by targeted agents, immunotherapy, and cellular therapies [4,5]. The five-year survival rate for multiple myeloma (MM) increased from approximately 30% in 1990 to 60% in 2019, while that for acute myeloid leukemia (AML) rose from 10% to 35% over the same period, reflecting significant advances including targeted therapies, immunotherapies, and optimized chemotherapy regimens [3]. Consequently, the growing population of long-term HSCT survivors has amplified the importance of comprehensive supportive care, positioning rehabilitation as an integral component of oncological management [6,7].

Hematopoietic stem cell transplantation (HSCT) remains a cornerstone of curative-intent treatment for selected HMs, including acute leukemias (ALs), lymphomas, and MM [7,8]. In 2023, 47,731 HSCTs were reported by 696 centers affiliated with the European Society for Blood and Marrow Transplantation (EBMT) across Europe and collaborating countries, including a 7.8% increase in allogeneic procedures and a 52.5% rise in chimeric antigen receptor (CAR) T-cell therapies compared with the preceding year [9]. Outcomes after HSCT have improved substantially over recent decades across major HM subtypes; in allogeneic-HSCT (allo-HSCT) for AML, a large registry study by the EBMT Acute Leukemia Working Party (ALWP), including more than 20,000 adults transplanted in first complete remission (CR1), demonstrated significantly improved overall survival (OS) and leukemia-free survival (LFS) in more recently transplanted patients compared with those transplanted in 1993–2002 [10]; this trend corroborated in older patients (aged ≥ 65 years), among whom a separate EBMT ALWP analysis reported an improvement in three-year OS from 37% to 49% between 2000 and 2021 [11]. MM represents the most common single indication for autologous-HSCT (auto-HSCT) [9]. Real-world data show that OS now exceeds 10 years in transplant-eligible patients, with current survival probabilities likely even higher given the recent introduction of anti-CD38 monoclonal antibodies and other novel agents [12]. However, cure of the underlying disease is not accompanied by full restoration of health: approximately two-thirds of HSCT survivors report at least one chronic health condition, and nearly one-fifth develop severe or life-threatening complications, with the cumulative incidence of chronic conditions continuing to rise over time from transplantation with no evidence of a plateau [13,14]. During and following HSCT—encompassing conditioning, the peri-transplant period, and early recovery—patients commonly experience substantial skeletal muscle wasting, reduced muscle strength, impaired cardiorespiratory endurance, and clinically meaningful declines in functional capacity and mobility [7,8,15]. Cancer-related fatigue is among the most prevalent and burdensome persistent symptoms following HSCT: clinically significant fatigue was reported by 83.9% of patients at one month, 68.4% at three months, and 64.7% at six months post-transplant, with most surviving patients continuing to experience persistent fatigue at one (62.3%), three (68.0%), and six years (63.8%) post-HSCT, with little improvement beyond three to six months post-transplant [16]. Its pathophysiology is multifactorial, encompassing anemia, systemic inflammation with pro-inflammatory cytokine dysregulation, direct effects of conditioning chemotherapy and total body irradiation, immunosuppressive therapy, sleep disturbance, and comorbid depression—factors that frequently co-occur and interact [17,18].

Rehabilitation is increasingly recognized as an essential component of care across all phases of treatment for patients with HMs, yet in the context of HSCT, standardized guidelines and structured care pathways remain largely absent [19]. The available evidence is characterized by considerable heterogeneity in intervention type, timing, intensity, and outcome assessment, limiting the generalizability of findings and the development of consensus recommendations [7]. Rehabilitation delivery in HSCT recipients is further complicated by the stringent infection-control measures required during the acute post-transplant period, as well as the highly variable and often rapidly changing clinical status of this population. Consequently, exercise programs must be individually tailored, initiated at low-to-moderate intensity, and adapted to institutional constraints and patient tolerance [19,20].

Physical activity improves endurance, muscle strength, treatment tolerability, and health-related quality of life (HRQoL) across multiple cancer types [21,22]. In patients with HMs specifically, exercise has been demonstrated to be both safe and feasible [23], with evidence suggesting that structured exercise programs may accelerate functional recovery beyond what is observed in non-exercising patients [24].

Aerobic training is established as a cornerstone of rehabilitation for patients with HMs, including HSCT recipients, with evidence from a systematic review and meta-analysis demonstrating significant improvements in functional capacity and lower limb strength [8]. In inpatient settings, it may serve as a practical bedside-compatible intervention that remains safe during intensive therapy [25]. Mohananey et al. highlight that aerobic exercises are feasible and scalable in clinical practice, owing to straightforward standardization, precise dosing of exercise intensity, and applicability in settings of restricted mobility [20].

Against this background, the present study aimed to evaluate the effects of a self-directed aerobic exercise program on a cycle ergometer in inpatients under isolation precautions related to HSCT.

## 2. Materials and Methods

A single-center prospective, non-randomized controlled interventional study with sequential group allocation was conducted at the Department and Clinic of Hematology, Cellular Therapies and Internal Medicine, Wroclaw Medical University, among patients eligible for HSCT. The study was conducted between 1 September 2022 and 31 October 2025. Data was collected during the period of inpatient isolation. The study group comprised 43 patients who participated in a self-directed aerobic exercise program consisting of aerobic exercise performed on a cycle ergometer. The control group consisted of 30 patients undergoing the standard transplantation procedure who did not participate in any form of physical exercise. No stratification by underlying diagnosis or transplant modality was applied, as the intervention was designed as a universal protocol adaptable to individual clinical status.

**Figure 1:**
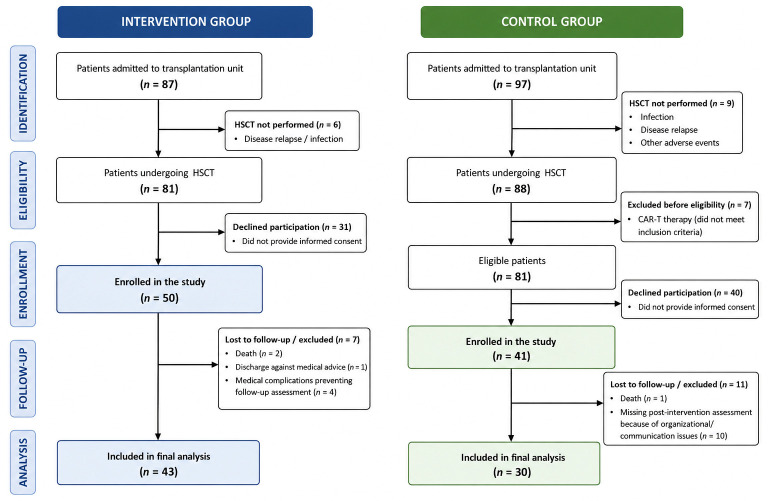
Flow diagram of participant recruitment, enrollment, follow-up, and final analysis.

Participants were recruited consecutively during hospitalization after meeting all eligibility criteria and providing written informed consent. This was a non-randomized controlled interventional study, and no random allocation procedure was performed. Group assignment was determined by the order of hospital admission. Consecutive eligible patients admitted during the project implementation period were enrolled in the intervention group. After completion of the intervention phase, consecutive eligible patients admitted to the same department and meeting the same eligibility criteria were enrolled in the control group. Both groups were recruited at the same hospital under comparable organizational conditions, following identical clinical procedures and with the involvement of the same medical staff. The reasons for exclusion from the final analysis are presented in Figure 1.

Inclusion criteria for the study group were age 18–75 years, a confirmed diagnosis of HM, eligibility for and scheduled to undergo HSCT, and signed written informed consent.

Exclusion criteria were cognitive impairment and any physical condition precluding safe exercise participation.

In the control group, engagement in any form of physical exercise during hospitalization was applied as an additional exclusion criterion.

Eligibility for HSCT at our institution required an ECOG performance status of ≤2; therefore, all participants enrolled in the study met this criterion.

The study protocol was approved by the Bioethics Committee of Wroclaw Medical University (No. KB-843/2021; 28 October 2021) and conducted in accordance with the Declaration of Helsinki. Written informed consent was obtained from all participants.

### 2.1. Assessment of Physical Performance

Physical performance was assessed using standardized functional tests—the 6-min walk test (6 MWT), the Timed Up and Go test (TUG), and the 30-s chair stand test (30 s-CST)—all of which were administered by trained physiotherapists. These tests have been established as valid, reliable, and clinically useful measures of exercise capacity, functional mobility, and lower-extremity muscle strength, including in oncology populations. Furthermore, 6 MWT and 30 s-CST outcomes have been shown to correlate with peak oxygen uptake (VO_2_peak), supporting their use as practical surrogate markers of physical performance in clinical settings, particularly when formal cardiopulmonary exercise testing is not feasible [26,27].

Functional assessments were performed at two time points: baseline (day 2 or 3 after hospital admission) and follow-up (after completion of treatment, either on the day preceding discharge or on the day of discharge).

The 6 MWT was conducted indoors on a flat 30-m corridor. Participants were instructed to walk as far as possible within 6 min at a self-selected walking pace, without running or jogging. At each end of the corridor, they turned and continued walking while maintaining the continuity of movement. Blood pressure (BP) and heart rate (HR) were measured in the seated position immediately before and immediately after completion of the 6 MWT. No BP or HR measurements were performed during the TUG or the 30 s-CST. If necessary, participants were allowed to slow down or rest in a seated position during the test; however, the stopwatch was not stopped. The total distance covered was recorded in meters.

The TUG evaluated a sequence of tasks relevant to independent mobility, including rising from a chair, walking 3 m, turning around, returning to the chair, and sitting down. The outcome measure was the total time required to complete the sequence, expressed in seconds. Participants started from a seated position. On the command “start,” they stood up, walked the designated 3-m distance as quickly as possible, turned, returned to the chair, and resumed the initial seated position, at which point timing was stopped. The test was required to be completed without loss of balance.

The 30 s-CST was performed from an unsupported seated position. Participants were instructed to keep their arms crossed over the chest. On the command “start,” they performed as many full sit-to-stand repetitions as possible within 30 s, achieving full upright extension and returning to the seated position each time. The examiner recorded the number of correctly completed full repetitions. Before the formal assessment, each participant performed 2–3 practice repetitions on the same day, immediately followed by the formal test.

### 2.2. Intervention Protocol

Patients enrolled in the intervention group participated in a self-directed aerobic exercise program performed without continuous supervision. Exercise sessions were performed using an ANTAR cycle ergometer (model AT51110; ANTAR Sp. J., Warsaw, Poland), registered as a medical device, with the resistance set at the lowest available level throughout the intervention period. At study entry, an individual target training HR was established for each participant as 60% of the age-predicted maximum HR, calculated as (220 − age) × 0.60 beats per min [28]. Heart rate was self-monitored by participants throughout each exercise session using a pulse oximeter. Participants were instructed to adjust their pedaling cadence to maintain their heart rate as close as possible to the prescribed target of approximately 60% of the age-predicted maximum heart rate.

Before commencing the exercise program, each participant received individualized instruction from a physiotherapist regarding the training protocol. In addition, participants had unlimited access to an instructional video demonstrating the correct exercise technique and training procedures, which they could consult throughout the intervention. The exercise sessions were then performed independently by the participants without continuous supervision.

Training was performed in a seated position on a chair with back support. The cycle ergometer was placed against a wall for stabilization, and the chair was positioned so that participants could comfortably extend their knees during pedaling. Participants exercised with their back supported and their hands resting on their thighs, while their feet were securely positioned on the pedals.

On the first day of training, participants performed 5 min of continuous forward pedaling. The exercise duration was increased by 5 min every 3 days until 40 min per day was reached. Participants were instructed to perform the training once daily throughout hospitalization.

Prespecified criteria for session discontinuation included the occurrence of sudden severe pain, chest pain, dyspnea, headache, dizziness, presyncope, nausea, and a paradoxical decrease or a sudden increase in HR or BP during exercise.

All participants maintained a daily exercise log documenting completion of the prescribed exercise program and selected patient-reported observations. Heart rate was self-monitored throughout each exercise session using a pulse oximeter, and adherence to the prescribed exercise program was periodically verified by the ward physiotherapist during routine inpatient visits. Patients were classified into the intervention group if they achieved at least 75% adherence to the exercise program, as predefined a priori in the study protocol. In the present study, all participants included in the final analysis met this predefined adherence criterion.

The exercise program was performed daily throughout the inpatient isolation period, from study enrollment until hospital discharge. The duration of the intervention corresponded to the length of inpatient isolation, with a mean duration of 30.82 ± 9.75 days.

For the purpose of this study, the term “self-directed aerobic exercise program” refers to an intervention in which participants exercised independently after individualized instruction, without continuous supervision by a physiotherapist during each exercise session.

### 2.3. Statistical Analysis

Statistical analyses were conducted using Statistica v13.3 (StatSoft, Kraków, Poland). The normality of data distribution was assessed using the Shapiro–Wilk test. Variables with a normal distribution are presented as mean ± standard deviation (SD), whereas variables with a non-normal distribution are presented as median and interquartile range (IQR). Categorical variables are reported as counts and percentages. Between-group comparisons were performed using the independent-samples Student’s *t*-test for normally distributed continuous variables, the Mann–Whitney U test for non-normally distributed continuous or ordinal variables, and the chi-square test for categorical variables.

To evaluate changes over time and differences between groups, a two-way repeated-measures analysis of variance (rm-ANOVA) was performed, with time (pre- vs. post-intervention) as the within-subjects factor and group (study vs. control) as the between-subjects factor. The assumption of sphericity was considered fulfilled because only two time points were analyzed; therefore, Mauchly’s test of sphericity was not applicable. The homogeneity of variances between groups was assessed using Levene’s test.

In addition to F-values and *p*-values, partial eta squared (ηp^2^) was reported as a measure of effect size. Mean changes (post-intervention minus pre-intervention values) with 95% confidence intervals (95% CI) were calculated to provide estimates of the magnitude and precision of treatment effects. Post hoc pairwise comparisons were performed with Bonferroni correction for multiple comparisons [29]. Statistical significance was set at *p* < 0.05. Observed statistical power was calculated based on the obtained effect sizes and sample size.

## 3. Results

A total of 73 patients were included in the study. Forty-three participants comprised the intervention group, whereas 30 comprised the control group. The mean age was 53.48 years in the intervention group and 55.26 years in the control group, whereas the mean Body Mass Index (BMI) was 27.13 kg/m^2^ and 26.78 kg/m^2^, respectively.

Patients were classified into six diagnostic subgroups according to the underlying hematologic disorder. In the intervention group, these included ALs, comprising AML and acute lymphoblastic leukemia (ALL) (*n* = 13); myelodysplastic syndromes (MDS) (*n* = 1); myeloproliferative neoplasms (MPN) (*n* = 3); lymphomas, including Hodgkin lymphoma (HL), diffuse large B-cell lymphoma (DLBCL), follicular lymphoma (FL), and peripheral t-cell lymphoma (PTCL) (*n* = 9); MM (*n* = 13); and other diagnoses, including aplastic anemia (AA), chronic myelomonocytic leukemia (CMML), and chronic neutrophilic leukemia (CNL) (*n* = 4). The corresponding categories in the control group were ALs, including AML and ALL (*n* = 10); MDS (*n* = 4); MPN (*n* = 1); lymphomas, including HL, DLBCL, and mantle cell lymphoma (MCL) (*n* = 4); MM (*n* = 7); and other diagnoses, including AA, CMML, and plasma cell leukemia (PCL) (*n* = 4). Regarding transplant type, 23 patients in the intervention group and 21 in the control group underwent allogeneic-HSCT (allo-HSCT), whereas 20 and 9 patients, respectively, underwent autologous-HSCT (auto-HSCT). No significant between-group differences were observed with respect to age, sex, BMI, clinical characteristics, hemoglobin (Hb) levels, ECOG performance status, CIRS, HCT-CI, or hospitalization-related parameters, including days from admission to discharge and days from HSCT to discharge (*p* > 0.05; Table 1).

Rm-ANOVA revealed a significant group-by-time interaction for the TUG (F(1,71) = 10.35; *p* = 0.001,ηp^2^ = 0.13) and the 30 s-CST (F(1,71) = 21.61; *p* < 0.0001, ηp^2^ = 0.23), with high statistical power in both cases (power > 0.8). No significant group-by-time interaction was found for the 6 MWT (F(1,71) = 2.01; *p* = 0.16, ηp^2^ = 0.03). Detailed findings are presented in Table 2.

In the 6 MWT, the distance covered at follow-up decreased in both groups, by 4.1% in the intervention group and by 10.5% in the control group. Post hoc analysis demonstrated a significant decline only in the control group (*p* = 0.017). For the TUG, completion time increased in both groups; however, the increase was greater in the control group (21.4%) than in the intervention group (4.9%). A significant pre–post difference was observed exclusively in the control group (*p* < 0.001). In the 30 s-CST, the number of repetitions increased by 5.7% in the intervention group, whereas a 14.8% decrease was observed in the control group. Post hoc analysis confirmed a significant deterioration in the control group (*p* < 0.001). Detailed results are presented in Table 3.

## 4. Discussion

The findings of the present study indicate significant differences in the trajectory of functional changes, as assessed by the 6 MWT, the TUG, and the 30 s-CST, between the self-directed aerobic exercise group and the control group.

Regarding the 6 MWT, a reduction in the distance covered was observed in both groups. The decline was smaller in the intervention group (−4.1%) than in the control group (−10.5%). Although this difference did not reach statistical significance in the group-by-time interaction model, suggesting that the temporal changes in exercise capacity were comparable between the groups, the numerically smaller decline in the intervention group may suggest a smaller decline in physical performance; however, this difference did not reach statistical significance. It is possible that, during hospitalization, the impact of intensive treatment and other clinical factors outweighs the potential benefits of exercise intervention with respect to overall physical capacity. Performance in the 6 MWT is strongly determined by the patient’s general clinical status, including disease burden, anemia, and inflammatory markers [30]. Ferreira et al. demonstrated that among patients with inflammatory or chronic diseases, higher inflammatory marker levels and lower hemoglobin concentrations were independently associated with a shorter distance achieved in the 6 MWT [30]. An association between 6 MWT performance and health status indicators has also been demonstrated in populations with complex clinical profiles, including liver transplant recipients [31]. This underscores the importance of the test as a tool for assessing overall physical functioning rather than solely muscle performance or locomotor capacity [31].

Potiaumpai et al. conducted a randomized study in 35 patients undergoing HSCT. The intervention group participated in a multimodal walking program, whereas the control group received standard care. At the first assessment, performed 3–5 days after transplantation, a statistically significant reduction in 6 MWT distance was observed in both groups. At the second assessment, conducted 30 days after transplantation, the intervention group demonstrated a statistically significant improvement in 6 MWT performance, whereas no significant improvement was observed in the control group. Although neither group regained the pre-transplant 6 MWT distance, the authors emphasized that peritransplant exercise may be associated with better preservation of cardiorespiratory capacity following HSCT, compared with the absence of physical intervention [32].

Borham et al., in a randomized study, demonstrated that a 3-month exercise intervention significantly increased 6 MWT distance in women with active chronic graft-versus-host disease (cGvHD) after HSCT compared with women receiving routine care. A greater decrease in interleukin-6 (IL-6) levels was observed in the exercise group than in the control group. Although this difference did not reach statistical significance, the findings suggest that exercise-based interventions may attenuate systemic proinflammatory cytokine levels [33].

In a pilot study by Rothe et al. investigating the effects of cardiac rehabilitation in patients with lymphoma undergoing auto-HSCT, following a 3-week rehabilitation program, the 6 MWT distance recorded at 9 weeks post-HSCT was significantly greater than that recorded at 6 weeks post-HSCT (*p* < 0.001) and greater than the pre-transplant baseline value (*p* < 0.01) [34]. In contrast, the self-directed aerobic exercise intervention applied in the present study did not yield a significant improvement in 6 MWT performance.

Physical exercise may significantly improve 6 MWT performance, as demonstrated in the systematic reviews by Martín-Sánchez et al., Liang et al., Morales Rodríguez et al., and Abo et al. [8,21,35,36]. Exercise-based interventions have been shown to improve aerobic capacity and functional status after transplantation. It has been emphasized that the duration of the intervention is a key determinant of significant post-training improvements, with the most pronounced changes observed in programs lasting over 12 weeks [8]. In the present study, the intervention was limited to the inpatient isolation, which may explain the absence of statistically significant changes.

To better contextualize the physical status of our cohort, it should be emphasized that, to our knowledge, normative reference values for the 6 MWT, TUG, and 30 s-CST have not been established specifically for patients with hematological malignancies undergoing HSCT. Therefore, interpretation of physical performance in this population relies primarily on comparisons with healthy reference populations. In our previous study, patients qualified for HSCT demonstrated significantly lower exercise capacity and lower-extremity muscle performance than age- and sex-matched healthy adults, whereas functional mobility assessed using the TUG test remained comparable [15]. These observations are consistent with published normative data for healthy adults, which indicate greater 6 MWT distances and higher 30 s-CST performance than those observed in our cohort, while baseline TUG values remain within the expected range for adults of comparable age [37,38]. Together, these findings suggest that patients undergoing HSCT already present with impaired exercise capacity and lower-extremity functional performance before transplantation despite relatively preserved basic functional mobility.

Regarding functional mobility, as assessed by the TUG, the present study demonstrated a greater increase in test completion time in the control group (+21.4%) than in the intervention group (+4.9%), whereas significant pre–post differences were observed only in the control group. These findings may indicate that the self-directed aerobic exercise intervention was associated with a smaller decline in locomotor function relevant to activities of daily living.

Potiaumpai et al. reported a significant prolongation of TUG completion time in both groups—the intervention group, which performed aerobic training comprising multidirectional walking, and the control group receiving standard care [32].

In a prospective observational study evaluating balance and physical performance after transplantation in 30 patients undergoing allo-HSCT, a significant prolongation of TUG completion time (*p* < 0.01) was observed, together with significant reductions in handgrip strength, knee extensor strength, and 6 MWT distance (*p* < 0.01) [39]. Moreover, TUG performance was negatively correlated with muscle strength, the deterioration of which may impair balance in patients undergoing allo-HSCT [39]. The findings of the present study are partially consistent with these observations. Functional deterioration was observed in the control group, whereas no significant changes over time were noted in the intervention group. These findings suggest that an exercise intervention in the form of a self-directed aerobic exercise program may mitigate adverse functional changes associated with HSCT.

In the 30 s-CST, the number of repetitions increased by 5.7% in the intervention group, whereas a 14.8% decrease was observed in the control group. Post hoc analysis confirmed a significant deterioration in performance in the control group (*p* < 0.001). By contrast, the absence of significant pre-post differences in the intervention group (*p* > 0.05) suggests preservation of functional capacity over time. The 30 s-CST is widely recognized as a valid and reliable measure of lower-extremity functional strength and physical performance [40].

Hacker et al. investigated the effects of resistance training on physical function in patients undergoing HSCT compared with standard care. In both the exercise and control groups, temporal changes in 30 s-CST performance were not statistically significant. Lower-extremity muscle strength declined after transplantation but returned to approximately pre-transplant levels after 6 weeks. The authors concluded that muscle strength was influenced primarily by time elapsed since transplantation rather than by participation in the intervention [41].

Potiaumpai et al. compared an exercise group with a standard-care group and showed that, at more than 100 days after transplantation, patients exposed to exercise had recovered and, in some cases, even improved beyond their pre-treatment level of physical performance, as assessed by the 6 MWT, 30 s-CST, and TUG. In contrast, patients who did not receive the intervention failed to return to baseline performance in any of these tests [24]. As the intervention in that study was delivered before, during, and after transplantation, these findings suggest that a long-term exercise program may be more effective than shorter or exclusively peritransplant interventions. This interpretation is consistent with the meta-analysis by Martín-Sánchez et al. [8], which demonstrated a moderate, statistically significant effect of physical exercise on functional capacity assessed by the 6 MWT (effect size approximately 0.43), corresponding to a clinically meaningful increase in walking distance of approximately 30–40 m. In addition, exercise training significantly improved lower-extremity muscle strength, supporting the interpretation of the present TUG and 30 s-CST results, both of which depend substantially on lower-limb muscle function. In contrast, the effects of exercise on fatigue and QoL were less consistent, likely reflecting heterogeneity in intervention protocols and duration [8].

Although the present study evaluated exclusively aerobic exercise, the available evidence indicates that resistance exercise may also contribute to preserving muscle strength and functional performance in patients undergoing HSCT [8,21,41]. Therefore, aerobic and resistance exercise should be regarded as complementary rather than competing rehabilitation strategies in this patient population.

There is growing evidence that exercise-based interventions, including self-directed rehabilitation, represent an effective supportive component of care in patients undergoing HSCT [19,21]. Favorable effects have been reported on exercise capacity, as measured by the 6 MWT [7,8], upper- and lower-limb muscle strength [8,21], and functional performance assessed with the 30 s-CST and TUG [7,21]. Exercise interventions may also mitigate the decline in physical performance during the peritransplant period [20,24].

The present study adds to the current evidence in several important ways. Previous clinical studies and systematic reviews have generally shown that exercise during HSCT is safe and may provide clinically meaningful benefits [6,7,8,21]. However, many published interventions have involved multimodal exercise programmes with varying degrees of supervision and frequently extended beyond the inpatient transplantation period [6,7,8,21,24]. In contrast, the present study evaluated a structured self-directed aerobic exercise programme implemented throughout the acute inpatient phase of HSCT, representing one of the most clinically demanding stages of treatment. Rather than producing measurable improvements in physical performance during hospitalization, our findings suggest that the principal value of this pragmatic intervention may lie in preserving functional mobility and limiting functional decline. Because the programme required only limited physiotherapist supervision and simple equipment, it may represent a feasible strategy that could be incorporated into routine inpatient HSCT care. Therefore, the present findings complement the existing literature by providing further evidence supporting the feasibility and potential clinical value of structured self-rehabilitation during transplantation.

### 4.1. Limitations

This study has several limitations. The relatively small sample size precluded subgroup analyses according to underlying diagnosis and transplant type. Moreover, the non-randomized design and the sequential recruitment of participants may have introduced selection bias and residual confounding. Furthermore, although the difference was not statistically significant, the higher proportion of allogeneic HSCT recipients in the control group may have acted as a potential confounding factor and should be considered when interpreting the results. Although both groups were recruited in the same center under comparable clinical conditions, unmeasured patient- or disease-related factors may have influenced the observed outcomes. In addition, although participants self-monitored heart rate during exercise, maintained daily exercise logs, and adherence was periodically verified by the ward physiotherapist, detailed session-by-session quantitative data on exercise execution were not prospectively collected in a form suitable for quantitative analysis. Therefore, some variability in the implementation of the exercise protocol between participants cannot be excluded. Moreover, the observation period was restricted to the inpatient isolation phase, which precluded evaluation of the long-term effects of the intervention in this patient population.

### 4.2. Future Directions

The limitations of the present study point to several areas for future research. First, larger studies are needed, with analyses stratified by underlying diagnosis and transplant modality. Second, future protocols should incorporate adherence monitoring to assess its relationship with outcome variability. Third, longer follow-up is warranted to determine the long-term effects of the intervention. Finally, future studies should include additional patient-reported and clinical outcomes, particularly validated measures of fatigue and quality of life, together with complementary assessments, such as hand-grip strength, cardiorespiratory fitness (e.g., VO_2_max), and body composition analysis using bioelectrical impedance vector analysis (BIVA), to provide a more comprehensive evaluation of functional recovery following HSCT.

## 5. Conclusions

The present findings suggest that self-directed aerobic exercise performed during inpatient isolation may help preserve functional mobility and mitigate deterioration in physical performance in patients undergoing HSCT. In particular, the observed effects in mobility-based functional tests indicate a potential role for a cycle ergometer-based self- directed aerobic exercise program in reducing the functional consequences of transplantation-related deconditioning. These results support the inclusion of structured, feasible exercise strategies as a supportive element of inpatient care in this population. Further research should evaluate the potential benefits of combining aerobic and resistance exercise in patients undergoing HSCT.

## Figures and Tables

**Table 1:** Baseline characteristics of the study groups.

Variable	Study Group	Control Group	*p*-Value *
*n*	43	30	-
Age, [years]; mean (SD)	53.48; 13.99	55.26; 13.40	0.81
BMI, [kg/m^2^]; mean (SD)	27.13; 4.60	26.78; 5.35	0.36
Gender, *n* (%)			0.83
Female	24; 55.81%	16; 53.33%	
Male	19; 44.19%	14; 46.67%	
Diagnosis *n* (%)			0.45
MM	13; 30.23%	7; 23.33%	
ALs	13; 30.23%	10; 33.33%	
MPN	3; 6.98%	1; 3.33%	
Lymphomas	9; 20.93%	4; 13.33%	
MDS	1; 2.33%	4; 13.33%	
Other	4; 9.30%	4; 13.33%	
Type of HSCT, *n* (%)			0.16
auto	20; 46.51%	9; 30.00%	
allo	23; 53.49%	21; 70.00%	
Hb [g/dL], mean (SD)	11.24; 2.27	10.97; 2.27	0.61
Days from admission to discharge, Me (IQR)	29.00; 11.00	30.00; 13.00	0.77
Days from HSCT to discharge, Me (IQR)	21.00; 8.00	12.00; 9.00	0.70
ECOG, *n* (%)			0.37
0	23;53.49%	13; 43.33%	
1	20; 46.51%	16; 53.33%	
2	0; 0.00%	1; 3.33%	
CIRS, *n* (%)			0.51
>6	13; 30.23%	7; 23.33%	
≤ 6	30; 69.77%	23; 76.67%	
HCT CI, *n* (%)			0.20
0—Low risk (0 points)	26; 60.47%	24; 80.00%	
1—Intermediate risk (1–2 points)	12; 27.91%	4; 13.33%	
2—High risk (≥3 points)	5; 11.63%	2; 6.67%	

Abbreviations: BMI, body mass index; SD, standard deviation; Me, Median; IQR, interquartile range; MM, multiple myeloma; ALs, acute leukemias; MPN, myeloproliferative neoplasms; MDS, myelodysplastic syndromes; HSCT, hematopoietic stem cell transplantation; auto, autologous hematopoietic stem cell transplantation; allo, allogeneic hematopoietic stem cell transplantation; n, number; Hb, Hemoglobin; ECOG, Eastern Cooperative Oncology Group performance status; CIRS, Cumulative Illness Rating Scale; HCT-CI, Hematopoietic Cell Transplantation–Specific Comorbidity Index. * *p*-values were calculated using Student’s *t* test or the Mann–Whitney U or the chi-square test, as appropriate.

**Table 2:** Results of the repeated-measures analysis of variance.

ANOVA														
	Pre		Post				Repetition				Repetition × Group			
Group	Mean	SD	Mean	SD	Δ	Δ 95% CI	F(1,71)	*p*	Partial η^2^	Test Power	F(1,71)	*p*	Partial η^2^	Test Power
The 6 MWT [m]														
SG	431.53	±86.70	413.94	±97.73	−17.59	−39.50;4.32	11.04	0.001	0.13	0.90	2.00	0.16	0.03	0.28
CG	418.67	±78.17	374.90	±103.73	−43.76	−75.19;−11.61								
The TUG [s]														
SG	6.57	±1.45	6.89	±1.94	0.32	−0.05;0.68	25.76	<0.0001	0.27	0.99	10.35	0.001	0.13	0.88
CG	6.65	±1.69	8.07	±2.49	1.42	0.77;2.07								
The 30 s-CST [reps]														
SG	11.84	±3.49	12.52	±3.25	0.67	0.06;1.29	4.75	0.03	0.06	0.57	21.60	<0.0001	0.23	0.99
CG	12.56	±3.03	10.70	±3.48	−187	−2.86;−0.87								

Abbreviations: ANOVA, analysis of variance; SD, standard deviation; Δ, mean change; Δ 95% CI, 95% CI of mean change; 6 MWT, 6-min walk test; TUG, Timed Up and Go test; 30 s-CST, 30-s chair stand test; SG, study group; CG, control group; Pre, baseline assessment; Post, post-intervention assessment; F, F statistic; *p*, *p*-value; partial η^2^, partial eta squared; m, meters; s, seconds; reps, repetitions.

**Table 3:** Comparison of mean test values between consecutive within-group assessments using the Bonferroni post hoc test.

Outcome	Comparison	*p*-Value
6 MWT [m]	Study pre–post	0.85
	Control pre–post	0.017
	Study post vs. Control post	0.47
TUG [s]	Study pre–post	0.90
	Control pre–post	<0.001
	Study post vs. Control post	0.06
30 s-CST [reps]	Study pre–post	0.35
	Control pre–post	<0.001
	Study post vs. Control post	0.14

Abbreviations: 6 MWT, 6-min walk test; m, meters; TUG, Timed Up and Go test; s, seconds; 30 s-CST, 30-s chair stand test; reps, repetitions; pre, baseline assessment; post, post-intervention assessment.

## Data Availability

The original contributions presented in this study are included in the article. Further inquiries can be directed to the corresponding author.
